# Technical, Tactical, and Time–Motion Match Profiles of the Forwards, Midfielders, and Defenders of a Men’s Football Serie A Team

**DOI:** 10.3390/sports13020028

**Published:** 2025-01-21

**Authors:** Rocco Perrotta, Alexandru Nicolae Ungureanu, Domenico Cherubini, Paolo Riccardo Brustio, Corrado Lupo

**Affiliations:** 1 Facultad de Deporte, Catholic University of Murcia (UCAM), 30107 Murcia, Spain; rperrotta@alu.ucam.edu (R.P.); dcherubini@ucam.edu (D.C.); 2 Empoli Football Club, 50053 Empoli, Italy; 3 NeuroMuscularFunction|Research Group, School of Exercise and Sport Sciences, SUISM, University of Turin, 10126 Turin, Italy; alexandru.ungureanu@unito.it (A.N.U.); paoloriccardo.brustio@unito.it (P.R.B.); 4 Department of Life Sciences and Systems Biology, University of Turin, 10126 Turin, Italy; 5 Department of Clinical and Biological Sciences, University of Turin, 10126 Turin, Italy; 6 Department of Medical Sciences, University of Turin, 10126 Turin, Italy

**Keywords:** soccer, performance analysis, tactical roles, key performance indicators, game profiles, integrated analysis

## Abstract

The present study aimed to verify the (1) differences between players’ roles in relation to technical and tactical and time–motion indicators, and the (2) relationships between individual time–motion and technical and tactical indicators for each role in a men’s Italian football Serie A team. A total of 227 performances were analyzed (28 players: 8 forwards, FWs; 11 midfielders, MDs; 9 defenders, DFs). Technical and tactical indicators, such as ball possession (played balls, successful passes, successful playing patterns, lost balls, ball possession time), offensive play (total and successful dribbles, crosses, assists), and shooting (total shots, shots on target) were obtained by means of Panini Digital (DigitalSoccer Project S.r.l). In addition, a time–motion analysis included the total distance, distances covered at intensities of 16.0–19.8 km/h, 19.8–25.2 km/h, and over 25.2 km/h, the average recovery time between metabolic power peaks, and burst occurrence, the latter of which was performed by means of a 18 Hz GPS device (GPexe Pro2 system tool) worn by the players. Results showed role-specific differences: MDs covered more distance, while DFs had better ball possession. MDs and DFs had more successful playing patterns, and MDs and FWs performed more dribbles and shots. Strong correlations (*p* < 0.01, ρ > 0.8) were found between bursts and assists for FWs, high-intensity running and ball possession for MDs, and distance, dribbling, and shots for DFs. These findings highlight the importance of individual and tailored training programs to optimize role-specific performance demands.

## 1. Introduction

Match analysis has been widely utilized to study the technical, tactical, and physical aspects of football performance, particularly over the past two to three decades, by the means of time–motion (TMA) notational analysis [1,2,3,4]. Recent studies have increasingly focused on technical and tactical aspects with a growing emphasis on both individual and team levels considering different styles of play [5,6], thanks to valid and reliable observational methods and technologies [7].

From the TMA perspective, several studies have focused on assessing players’ performance in top men’s football leagues, including the Brazilian Serie A [8], Spanish LaLiga [9], English Premier League [10,11,12], French Ligue 1 [13], German Bundesliga [14], and Italian Serie A [15], by the means of a wide range of technologies (e.g., video tracking, GPS, local positioning systems). These studies have analyzed various time–motion parameters, such as standing, walking, jogging, running, high-speed running, and sprinting, to compare different tactical roles within specific competition levels. For instance, Vigne et al. found that Serie A midfielders covered the greatest total distance and had more frequent displacements and sprints compared to players in other positions using a multi-camera tracking system [15].

A recent systematic review that examined various time–motion technologies, including semi-automated camera tracking, GPS, and microwave radio frequency systems, found that Serie A players had the highest average total distance (11.389 m) among the top leagues [16]. This review also highlighted significant role-specific differences: center backs generally covered the lowest total distances and had the least high-speed and very-high-speed activities, while wide midfielders had the highest total distances and exhibited the most high-speed and very-high-speed activities.

Further research on Serie A [17] using a semi-automatic tracking system reported that central and wide defenders covered less distance in high-speed and very-high-speed running compared to players in other positions. In addition, according to a recent review [18], the literature principally reported that MDs and external DFs cover greater total and high-speed distance than FWs or central DFs, and sprint distance was higher in external MDs than in all other positions. On the other hand, from the notational analysis perspective, performance in elite football was investigated exclusively from a technical and tactical point of view [19,20,21,22], including specific information such as competitive level, game location, quality of opposition, and match half. However, DFs and central MDs performed more passes than all other tactical roles according with the most of the studies [18].

In particular, Alberti et al. reported that goal scoring frequency was notably higher in the second half, especially in the last 15 min of matches, across the top four European leagues (English Premier League, French Ligue 1, Italian Serie A, and Spanish La Liga) [19]. Moreover, Rampinini et al. found that the top-ranked Serie A teams (those in the top five positions) were more active with the ball, achieving higher numbers in short passes, successful short passes, tackles, dribbling, shots, and shots on target compared to teams in the bottom five [20]. Similarly, Lago-Ballesteros and Lago-Peñas observed that lower-ranked teams in Spain’s top league required more shots to score and had fewer assists and less ball possession than higher-ranked teams [21]. More recently, Kubayi examined technical roles in European Championship qualifying matches, revealing that forwards, central midfielders, and wide midfielders produced more shots, passes, and crosses than players in other positions, while forwards were more likely to lose possession compared to central defenders [23].

Nevertheless, very few studies have combined TMA with technical and tactical performance metrics in an integrated match analysis perspective. Among these, Liu and colleagues [24] aimed to investigate the impact of high (HPBPT) and low percentage ball possession teams (LPBPT) on physical and technical–tactical performance indicators in the Chinese Football Super League, revealing that central defenders and fullbacks covered more high-intensity and sprint running distance in the high possession teams, while wide midfielders and forwards covered more high-intensity and sprint running distance in the low possession teams. Moreover, players in high ball possession teams showed superior technical performance, especially in attacking organization. In Europe, Modric and colleagues [25] examined UEFA Champions League technical and tactical performance differences between teams with high and low running performance and they found that the tactical outcomes were similar across teams with different total distances (TDs) and running intensities. However, teams with higher TDs and ball possession achieved more goal chances, shots, shots on target, passes, accurate passes, key passes, accurate key passes, crosses, successful high pressing, entries into the opponent’s box, and overall successful actions. In a follow-up study [26], the effects of ball possession percentages on physical and technical performance were analyzed. The study revealed that wide midfielders and forwards in low ball possession teams covered higher TDs, engaged in more low-intensity running, and achieved higher average speeds compared to their counterparts in high ball possession teams. Additionally, players in high ball possession teams had more passes and successful passes. Overall, these findings underscore that both physical and technical and tactical performances are strongly influenced by ball possession, with clear position-specific variations.

Continuing the effort to integrate physical and technical and tactical aspects, the most recent study by Bradley [27] aimed to contextualize and benchmark the physical demands of teams in the FIFA World Cup Qatar 2022. Despite detailed data on distances covered at varying intensities and fluctuations across matches and halves, the results revealed notable correlations between the frequency of high-intensity runs and the key phases of play, such as defensive transitions, recoveries, and advancements into the final third of the field.

Among the studies reviewed, only one [24] specifically analyzed tactical roles, offering valuable insights into interpreting time–motion performance models and guiding individualized training. More specifically, for Italian Serie A, a recent study was able to highlight that that high-ranking teams exhibited a higher percentage of high-intensity accelerations compared to mid-ranking teams, and the 4-3-3 playing formation was associated with greater acceleration demands than other formations, particularly in high ranking teams [28]. Nevertheless, despite a progressive number of papers on football performance being published in recent years [18], there is a notable gap in research that combines TMA with technical and tactical performance in Serie A matches. Therefore, this study aimed to (1) examine the differences between roles (forwards, FWs; midfielders, MDs; defenders, DFs) regarding time–motion and technical–tactical indicators, and (2) explore the relationships between individual time–motion and technical–tactical indicators for players from a Serie A team.

## 2. Materials and Methods

### 2.1. Participants

A total of 28 players (8 FWs, age = 25.8 ± 5.4 years, stature = 183.8 ± 5.9 cm, weight = 75.1 ± 4.6 kg; 11 MDs, age = 25.5 ± 3.5 years, stature = 178.8 ± 4.9 cm, weight = 71.9 ± 5.8 kg; 9 DFs, age = 25.1 ± 2.9 years, stature = 185.1 ± 5.2 cm, weight = 73.8 ± 4.9 kg) of a men’s Serie A football team (Empoli Football Club; ranked in the 14th final position over a total of 20 teams, i.e., 3rd ranking quartile) performed a total of 227 individual match performances (82 ± 12min) during the 33 team matches of the 2022–2023 season. This sample includes all matches of the season, except for five away games where the stadium infrastructure interfered with the GPS signal recording. The goalkeeper has been excluded in this study because of availability of different parameters for this role with respect to those of the others. For inclusion, players were required to participate in at least five matches, each lasting more than 45 min, with complete TMA and technical–tactical data available. As a result, each player included in the analysis completed an average of 12 ± 8 matches during the season (range: 5–30), with FWs averaging 8 ± 6 matches (range: 5–18), MDs averaging 12 ± 8 matches (range: 5–25), and DFs averaging 15 ± 9 matches (range: 5–30). Each player participating in the study signed a consent form for the use of data. The study was approved by the local ethics committee of the University of Turin (Protocol #0619909).

### 2.2. Methods

The present study was developed by means of individual players’ time–motion, and technical and tactical analyses (data were accessed for research purposes on 15 October 2024). The TMA data were obtained through the GPexe Pro2 system tool (Exelio S.r.l., Udine, Italy), a high-frequency 18 Hz GPS device approved by FIFA for official competitions which tracks players’ position over time, along with all respective derivatives such as speed, acceleration, and deceleration. Inertial units provide information on impacts, jumps, and running directions. All data were stored in the cloud for quick download. The device, placed in a sleeveless vest worn by each player during matches, also accounted for individual time–motion parameters like bursts, with reference values regularly updated, including data from training sessions. The technical and tactical data for each player in each match were provided by Panini Digital (DigitalSoccer Project S.r.l., Modena, Italy), offering over 500 performance indicators with a margin of error of <3%.

### 2.3. Procedures

The following time–motion indicators were considered for each player recruited for the study:

- Total distance (TD) expressed in meters;
- Distances covered at 16.0–19.8 km/h (Z2) expressed in meters;
- Distances covered at 19.8–25.2 km/h (Z3) expressed in meters;
- Distances covered at >25.2 km/h (Z4) expressed in meters;
- Average duration of the match periods occurring between patterns performed with a >20 W/Kg power peaks (MPErec);
- Occurrence of burst performed at >80% individual maximal acceleration (bursts).

The occurrences of the following technical and tactical indicators (grouped into ball possession, offensive play, and shooting indicators) were considered for each player.

The ball possession indicators are as follows:

- Played balls (i.e., individual possessions of the ball);
- Successful passes (i.e., passes also characterized by maintaining the team’s ball possession);
- Successful playing patterns (i.e., offensive patterns which determine an offensive advantage with respect to the opponents’ defense arrangement);
- Lost balls (i.e., lost balls due to a mistaken pass or an opponent stolen ball).

The times of ball possession (i.e., sum of ball possession pattern within an entire match) were recorded too.

The indicators of searching for advantageous opportunities were the following:

- Total and successful (and successful/total) dribbling (i.e., attempt to overtake another player with possession of the ball);
- Total and successful (and successful/total) crosses (i.e., airborne delivery of the ball into the opponent’s penalty area);
- Total and successful (and successful/total) assists (i.e., final pass or cross leading to the recipient of the ball scoring a goal).

The shooting indicator was as follows:

- Total shots and shots towards goal (shots towards goal/total shots).

### 2.4. Statistical Analysis

After considering the above-mentioned inclusion criteria, each player’s value has been standardized according to a conventional match duration (i.e., 90 min). Then, the mean values of each player for each TMA and technical and tactical indicator were calculated for statistical analyses. Nonparametric approach tests were applied. In particular, Kruskal–Wallis and Mann–Whitney tests were considered to compare the three analyzed tactical roles (aim 1), also calculating effect sizes (ESs) [29] to provide meaningful analysis for significant comparisons, considering ≤0.2, 0.6, 1.2, and >1.2 as trivial, small, moderate, and large ES, respectively [30]. In addition, for the relationships between time–motion and technical and tactical indicators, Spearman correlations (ρ) were applied for each specific role (aim 2). All analyses were conducted using the statistical package SPSS (version 29; IBM SPSS Statistics for Windows, Armonk, NY, USA), and the criterion for significance was set at a 0.05 alpha level.

## 3. Results

**Figure 1:**
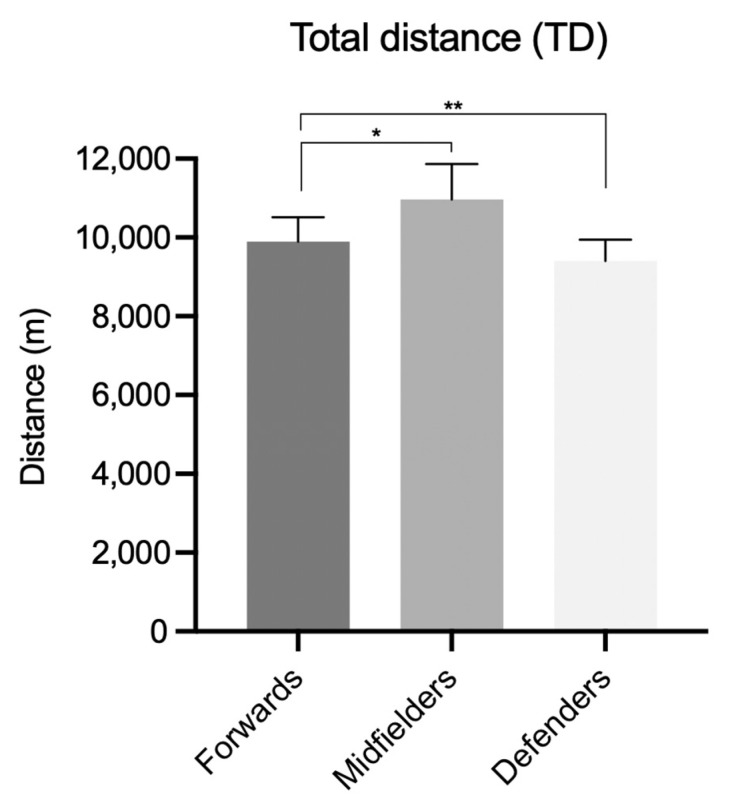
Total distance (TD) in relation to the three observed tactical roles (*, *p* < 0.05; **, *p* < 0.01).

For TMA indicators, the average team values were 10.158 ± 981 m for TD, with 1.058 ± 295 m, 611 ± 178 m, and 160 ± 71 m performed in the Z2, Z3, and Z4 speed zones, respectively; MPErec was 35.5 ± 6.7 s, whereas the mean occurrence of bursts was 25.2 ± 7.8. Among tactical roles, differences emerged for TD (*p* = 0.002), with higher MD values than FW (*p* = 0.027) and DF (*p* < 0.01) (Figure 1).

**Figure 2:**
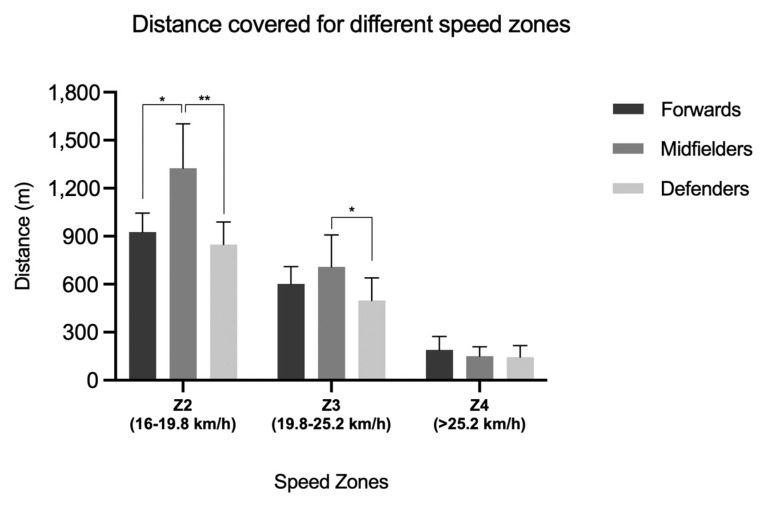
Distance at Z2, Z3, and Z4 speed zones, in relation to the three observed tactical roles (*, *p* < 0.05; **, *p* < 0.01).

Considering the specific observed speed zones, differences emerged for Z2 (*p* = 0.002), with specific differences between MDs and FWs (*p* = 0.012) and DFs (*p* < 0.01); and for Z3 (*p* = 0.045), with differences between MDs and DFs (*p* = 0.013); whereas no effect between roles emerged for Z3 (Figure 2), MPErec, or bursts.

From the notational analysis perspective (Table 1), DFs reported higher values than MDs and FWs, as well as MDs with respect to FWs, for played balls (main effect: *p* < 0.01; DF-MD: *p* = 0.028; DF-FW: *p* < 0.01; MD-FW: *p* = 0.015), successful passes (main effect: *p* < 0.01; DF-MD: *p* = 0.015; DF-FW: *p* < 0.01; MD-FW: *p* = 0.015). For successful playing pattern (main effect: *p* = 0.002), FWs reported lower values than MDs (*p* = 0.007) and DFs (*p* < 0.01). Moreover, DFs reported lower total dribbling (main effect: *p* = 0.036) than MDs (*p* = 0.039) and FWs (*p* = 0.018) and total assists (main effect: *p* = 0.013) only with respect MDs (*p* = 0.003). Then, DFs reported lower total shots (main effect: *p* = 0.01) and shots towards goal (main effect: *p* = 0.032) than MDs (*p* = 0.05, *p* = 0.034) and FWs (*p* = 0.003, *p* = 0.016). The time of ball possession was different between all roles (main effect: *p* < 0.01; DF-MD: *p* = 0.01; DF-FW: *p* < 0.01; MD-FW: *p* = 0.042).

**Figure 3:**
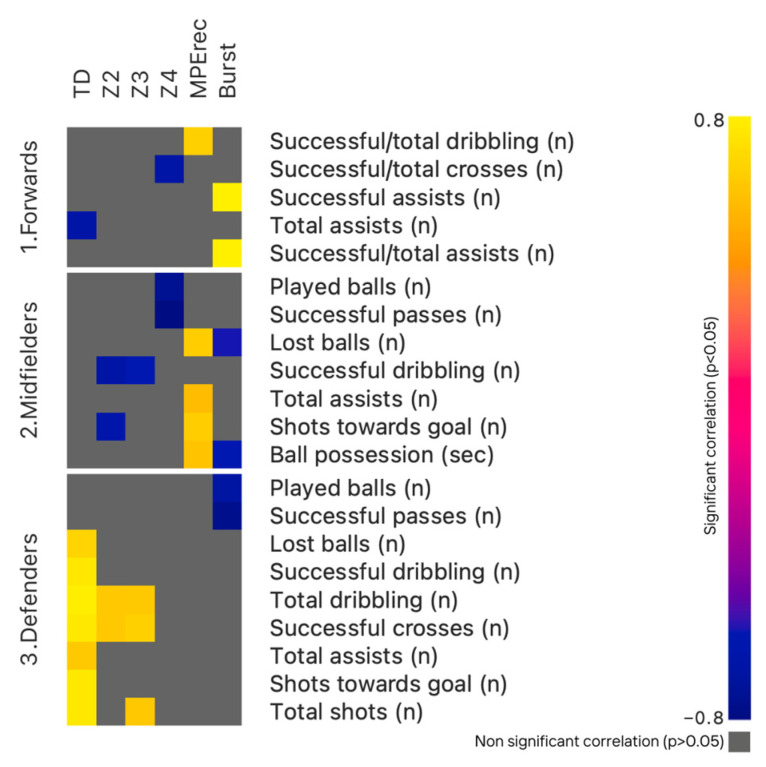
Relationships between TMA and the technical and tactical indicators for FWs, MDs, and DFs.

Finally, the relationships between TMA and the technical and tactical indicators for FWs, MDs, and DFs are shown in Figure 3 and detailed in Supplementary Materials (Table S1 for FWs, Table S2 for MDs, and Table S3 for DFs).

## 4. Discussion

This study aimed to examine potential differences in time–motion and technical and tactical factors across various tactical roles in players from a third quartile Serie A team during official matches. Additionally, it analyzed the relationships between time–motion and technical and tactical indicators for the same players to better understand the match profiles specific to each role. The main findings of this study revealed that the match profiles of these Serie A players are strongly influenced by their tactical roles, with distinct patterns (with differences generally associated with moderate effect size) and unique relationships between time–motion data and technical–tactical factors.

For time–motion factors, the highest TD overall (Figure 1), and in both Z2 and Z3 speed zones (Figure 2), was recorded for MDs, in line with previous studies [15,16,17] although with slightly lower absolute values compared to the French Ligue first division [31] and Premier League [11]. Also, for the other TMA indicators (Z4, MPErec, bursts), MD was distinguished with respect to other roles, according with previous analyses on football matches [15,16,17], whereas this is not confirmed in the present study. As a consequence, these differences, with respect to the results reported in the literature, may be attributed to role performance peaks, which could be less pronounced in teams ranked in the third quartile. In fact, team ranking, seasonal objectives, and specific players’ skills could substantially influence the team’s tactical approach (e.g., defensive approach more associated with third/fourth than first/second quartile team ranking; offensive approach more associated with first/second than third/fourth quartile team ranking) [32,33]. For this reason, the name of the club, season, and final quartile team ranking have been expressly reported in this study to provide a valuable reference for eventual and future studies on technical and tactical performance analyses regarding coherent or different competitive circumstances.

In terms of technical and tactical factors, DFs are mainly characterized by a playing profile focused on maintaining ball possession. In fact, DFs reported higher values than MDs and FWs (and MDs with respect to FWs) for the majority of ball possession indicators (e.g., played balls, successful passes, successful playing pattern, and time of ball possession). In particular, DFs reported higher frequencies of successful passes compared to both MDs and FWs, which was in line with a series of previous studies on elite football [18], but in contrast with findings from a previous study on the French Ligue first division, where central defenders had the lowest values for this indicator [31]. Therefore, for these performance factors, the above speculated effect determined by teams’ ranking, or playing styles associated with national championships, could explain the differences between the roles’ game profiles. However, even though this analysis was conducted without considering teams’ high- or low-ball possession [24,26] or comparisons with higher-ranking Serie A teams [20], it provides a clear representation of match performance profiles, where DFs, MDs, and FWs demonstrate a primary, intermediate, and minor importance in maintaining the ball possession, respectively.

For the “searching for advantageous opportunities” indicators (such as successful plays, and total and successful dribbles, crosses, and assists), MD proved to be the most influential role, executing more successful plays than FWs, and exceeding DFs in total dribbles. In line with the previous studies on international players [23], FWs displayed higher total dribbling numbers than DFs, while DFs exhibited more successful playing patterns than FWs, highlighting how DFs gained advantages through team-based actions (i.e., involving several teammates’ movements), and FWs, typically positioned farther from their own goal, often seek individual advantages through higher-risk actions like dribbling. Additionally, as expected given their playing zones, FWs and MDs recorded more total shots and shots on target than DFs. Interestingly, no significant differences were found between roles for lost balls or crosses, despite evidence at the international level that central and wide MDs typically exhibit higher values for these metrics. Similarly, cross success rates were similar among different roles, despite MDs contributing more significantly to overall assists than DFs, suggesting a more specialized distribution of playing responsibilities during offensive actions [23].

For the second aim of the present study, technical and tactical performance generally appears to be negatively correlated with high-intensity running, especially considering Z4 and bursts. In particular, the strongest negative correlations (*p* < 0.01; ρ < −0.8) were found between Z4 and both played balls and successful passes for MD, and between successful passes and bursts for DF, confirming findings of previous analyses [24], which suggested that these two high running intensities are more related to regaining possession than leading ball actions. In contrast, ball possession events seem to be more closely tied to lower (Z2) or moderate (TD) running intensities, as highlighted for DFs’ high positive correlations (*p* < 0.01; ρ > 0.8) between TD and total and successful dribbles, successful crosses, total, and shots towards goal. This results strongly suggest that lower running intensity enables DFs to perform dribbles, crosses, and shots, often from deeper positions, to initiate offensive plays or avoid losing possession, which could lead to an opposing counterattack given their often more defensive positioning. However, this general trend finds an exception in the FW profile, where a strong positive correlation emerged between bursts and both successful and total assists. Given the important role of FW not only in goal scoring but also in playmaking [34], it can be speculated that FWs use bursts to create an advantageous space from opposing defenders to deliver successful assists, also near the opponent’s goal and under high defensive pressure.

Generally, these findings offer valuable insights for elite football coaches and physical trainers. Technically, training sessions could be mainly characterized by workouts, where they should mainly consider shots and assists for FWs, successful plays and dribbles to create advantageous opportunities for MDs, and passes and ball possession skills for DFs. In addition, according to the reported relationships between technical and time–motion parameters, and the evident limits of technical success as running intensity increases, it emerges that the above-mentioned technical skills characterizing role performance could be also proposed by varying running intensities (or other game parameters such as type of opponents’ defense or court dimensions), thus trying to improve technical success and the running intensity ratio.

However, this study has some experimental limitations: i) the data were derived from a single team, with varying numbers of home and away matches; ii) to guarantee an adequate sample size, no specific distinctions were made between central and wide DFs or MDs, or between more or less advanced FW roles. Consequently, future research should aim to provide a more comprehensive understanding of time–motion and technical and tactical performance in Serie A, using larger player samples, evenly distributed between home and away matches, while considering team ranking even at higher positions.

## 5. Conclusions

The present study was focused on official Serie A matches played by players of a team (third ranking quartile) and revealed the following main distinct tactical role particularities:

- MDs are mainly characterized by non-maximal running distances compared to other roles;
- DFs excelled in most “ball possession” indicators (including played balls, successful passes, successful playing patterns, lost balls, and time of ball possession);
- MDs and FWs showed the highest values in “searching for playing advantage indicators” (such as total and successful dribbling, crosses, and assists);
- MDs and FWs recorded the highest values in “shots indicators” (total shots and shots on target);
- DFs and MDs are characterized by a negative association between the frequency of playing events and the highest intensity running categories (i.e., Z4 and bursts); conversely, FWs exhibited a positive correlation between bursts and assists, likely due to different team ball possession scenarios and offensive contexts [24].

Therefore, this study reinforces the idea that football performance in Serie A matches is strongly influenced by tactical roles, providing specific and valuable implications for training workouts.

## Figures and Tables

**Table 1:** Means ± standard deviations, ranges (min–max), and significances (and effect size, ES) between roles, in relation to each technical and tactical indicator.

Ball Possession Indicators	Forwards	Midfielders	Defenders
Played balls (n)	28.88 ± 7.00 (21–43) * (ES = 0.71) ^¥¥^ (ES = 0.92)	42.92 ± 6.11 (36–55) ^¥^ (ES = 0.62)	56.48 ± 9.19 (45–71)
Successful passes (n)	14.51 ± 5.07 (8–22) * (ES = 0.72) ^¥¥^ (ES = 0.91)	26.08 ± 6.04 (18–38) ^¥^ (ES = 0.73)	40.19 ± 7.64 (30–50)
Successful playing patterns (n)	3.05 ± 1.63 (1–7) ** (ES = 0.71) ^¥¥^ (ES = 0.73)	6.14 ± 1.79 (4–10)	8.20 ± 3.38 (4–13)
Lost balls (n)	12.16 ± 4.09 (7–21)	11.39 ± 4.23 (6–23)	8.72 ± 3.71 (5–14)
Ball possession (s)	69.21 ± 27.00 (36–124) * (ES = 0.64) ^¥¥^ (ES = 0.91)	104.01 ± 20.04 (78–141) ^¥¥^ (ES = 0.73)	153.74 ± 23.85 (123–175)
Searching for advantageous opportunities indicators	Forwards	Midfielders	Defenders
Successful dribbling (n)	1.29 ± 0.27 (1–2)	1.45 ± 0.86 (0–3)	0.81 ± 0.64 (0–2)
Total dribbling (n)	2.26 ± 0.84 (1–4) ^¥^ (ES = 0.23)	2.06 ± 1.14 (0–4) ^¥^ (ES = 0.61)	1.13 ± 0.86 (0–3)
Successful/total dribbling (n)	0.75 ± 0.38 (0–1)	0.54 ± 0.42 (0–1)	0.41 ± 0.34 (0–1)
Successful crosses (n)	1.03 ± 0.77 (0–2)	0.96 ± 0.81 (0–2)	0.55 ± 0.56 (0–1)
Total crosses (n)	2.05 ± 1.33 (0–4)	2.42 ± 0.97 (1–4)	2.48 ± 2.38 (0–8)
Successful/total crosses (n)	0.45 ± 0.50 (0–1)	0.32 ± 0.34 (0–1)	0.15 ± 0.21 (0–1)
Successful assists (n)	0.04 ± 0.07 (0–1)	0.04 ± 0.07 (0–1)	0.02 ± 0.04 (0–1)
Total assists (n)	0.58 ± 0.30 (0–1) ** (ES = 0.52)	0.92 ± 0.35 (1–2)	0.40 ± 0.36 (0–1)
Successful/total assists (n)	0.06 ± 0.09 (0–1)	0.06 ± 0.12 (0–1)	0.06 ± 0.12 (0–1)
Shooting indicators	Forwards	Midfielders	Defenders
Shots towards goal (n)	0.88 ± 0.69 (0–2) ^¥^ (ES = 0.32)	0.61 ± 0.30 (0–1) ^¥^ (ES = 0.51)	0.29 ± 0.22 (0–1)
Total shots (n)	1.72 ± 1.04 (0–3) ^¥¥^ (ES = 0.42)	0.97 ± 0.52 (0–2) ^¥^ (ES = 0.51)	0.44 ± 0.44 (0–1)
Shots towards goal/total shots	0.69 ± 0.49 (0–1)	0.94 ± 0.29 (1–2)	0.69 ± 0.53 (0–2)

*, ^¥^, *p* < 0.05; **, ^¥¥^, *p* < 0.01; differences with respect to MD and DF, respectively.

## Data Availability

The data presented in this study are available on request to the corresponding author. The data are not publicly available due to ethical considerations.

## Supplementary Materials

The following supporting information can be downloaded at https://www.mdpi.com/article/10.3390/sports13020028/s1, Table S1: Spearman (ρ and significance values) correlations between TMA and technical and tactical indicators for forwards. Table S2: Spearman (ρ and significance values) correlations between TMA and technical and tactical indicators for midfielders. Table S3: Spearman (ρ and significance values) correlations between TMA and technical and tactical indicators for defenders.
