# Supplementary material for: Technical, Tactical, and Time–Motion Match Profiles of the Forwards, Midfielders, and Defenders of a Men’s Football Serie A Team
Source: Sports (Basel). 2025 Jan 21;13(2):28. doi: 10.3390/sports13020028 (PMC11860454; doi:10.3390/sports13020028)
Supplement: Supplementary file 1 [file sports-13-00028-s001.zip › Table S1.pdf]

Table S1. Spearman (Rho and significance values) correlations between TMA and technical and tactical indicators for forwards.

Indicators	Correlations	TD	Z2	Z3	Z4	MPErec	Burst
Played balls (n)	Rho	0.286	0.571	0.667	0.214	-0.214	0.132
	p	0.493	0.139	0.071	0.610	0.610	0.721
Successful passes (n)	Rho	0.371	0.611	0.443	0.168	-0.072	-0.120
	p	0.365	0.108	0.272	0.691	0.866	0.778
Successful playing patterns (n)	Rho	-0.095	0.143	0.119	0.190	0.214	-0.190
	p	0.823	0.736	0.779	0.651	0.610	0.651
Lost balls (n)	Rho	-0.190	-0.024	0.214	-0.143	-0.357	0.333
	p	0.651	0.955	0.610	0.736	0.385	0.420
Fouls committed (n)	Rho	0.108	-0.036	0.084	0.299	-0.168	0.018
	p	0.799	0.933	0.844	0.599	0.691	0.966
Fouls received (n)	Rho	-0.048	0.143	0.524	0.476	-0.452	0.048
	p	0.911	0.736	0.183	0.233	0.260	0.911
Successful dribbling (n)	Rho	0.072	-0.126	-0.378	0.306	0.613	0.072
	p	0.878	0.788	0.403	0.504	0.144	0.878
Total dribbling (n)	Rho	-0.286	0.107	-0.036	-0.143	0.036	-0.500
	p	0.535	0.819	0.939	0.760	0.939	0.253
Successful/total dribbling (n)	Rho	0.071	-0.119	-0.095	0.405	0.714*	-0.381
	p	0.867	0.779	0.823	0.320	**0.047**	0.352
Successful crosses (n)	Rho	0.253	0.217	-0.482	-0.566	0.349	-0.217
	p	0.545	0.606	0.227	0.143	0.396	0.606
Total crosses (n)	Rho	0.071	0.286	0.167	0.143	-0.048	-0.238
	p	0.867	0.493	0.693	0.736	0.911	0.570
Successful/total crosses (n)	Rho	0.317	0.268	-0.439	-0.708*	0.366	-0.512
	p	0.444	0.520	0.276	**0.050**	0.373	0.194
Successful assists (n)	Rho	0.027	-0.109	-0.027	-0.218	-0.682	0.873**
	p	0.949	0.797	0.949	0.604	0.062	**0.005**
Total assists (n)	Rho	-0.714*	-0.476	-0.19	-0.333	-0.571	0.071
	p	**0.047**	0.233	0.651	0.420	0.139	0.867
Successful/total assists (n)	Rho	0.027	-0.109	-0.027	-0.218	-0.682	0.873**
	p	0.949	0.797	0.949	0.604	0.062	**0.005**
Shots towards goal (n)	Rho	0.204	0.036	0.132	0.024	-0.096	-0.024
	p	0.629	0.933	0.756	0.955	0.821	0.955
Total shots (n)	Rho	0.167	0.048	-0.381	-0.357	0.333	-0.500
	p	0.693	0.911	0.352	0.385	0.420	0.207
Shots towards goal/total shots (n)	Rho	0.204	0.012	0.180	0.11	0.335	0.22
	p	0.629	0.978	0.670	0.82	0.417	0.52
Ball possession (sec)	Rho	0.190	0.452	0.452	0.357	0.024	-0.190
	p	0.651	0.260	0.260	0.385	0.955	0.651

*(p≤0.05),**(p≤0.01) significant correlations.
