# Supplementary material for: Technical, Tactical, and Time–Motion Match Profiles of the Forwards, Midfielders, and Defenders of a Men’s Football Serie A Team
Source: Sports (Basel). 2025 Jan 21;13(2):28. doi: 10.3390/sports13020028 (PMC11860454; doi:10.3390/sports13020028)
Supplement: Supplementary file 1 [file sports-13-00028-s001.zip › Table S2.pdf]

Table S2. Spearman (Rho and significance values) correlations between TMA and technical and tactical indicators for midfielders.

Indicators	Correlations	TD	Z2	Z3	Z4	MPErec	Burst
Played balls (n)	Rho	0.055	0.027	-0.391	-0.809**	0.273	-0.591
	p	0.873	0.937	0.235	**0.003**	0.417	0.056
Successful passes (n)	Rho	0.409	0.400	-0.173	-0.864**	-0.100	-0.309
	p	0.212	0.223	0.612	**<0.001**	0.770	0.355
Successful playing patterns (n)	Rho	-0.464	-0.418	-0.536	-0.309	0.491	-0.345
	p	0.151	0.201	0.089	0.355	0.125	0.298
Lost balls (n)	Rho	-0.301	-0.346	-0.383	-0.182	0.697*	-0.610*
	p	0.369	0.297	0.245	0.592	**0.017**	**0.046**
Fouls committed (n)	Rho	0.182	0.418	0.445	0.055	-0.300	0.227
	p	0.593	0.201	0.170	0.873	0.370	0.502
Fouls received (n)	Rho	-0.055	0.041	-0.014	0.128	0.178	-0.351
	p	0.873	0.905	0.968	0.709	0.601	0.290
Successful dribbling (n)	Rho	-0.524	-0.715*	-0.620*	0.159	0.579	-0.451
	p	0.098	**0.013**	**0.042**	0.640	0.062	0.164
Total dribbling (n)	Rho	-0.273	-0.418	-0.082	0.418	0.155	0.182
	p	0.417	0.201	0.811	0.201	0.650	0.593
Successful/total dribbling (n)	Rho	-0.497	-0.642	-0.556	-0.105	0.588	-0.269
	p	0.120	0.033	0.076	0.759	0.057	0.424
Successful crosses (n)	Rho	0.055	0.138	0.138	0.202	0.055	-0.257
	p	0.872	0.687	0.687	0.552	0.872	0.446
Total crosses (n)	Rho	-0.456	-0.342	0.050	0.460	0.465	0.128
	p	0.159	0.304	0.884	0.154	0.150	0.709
Successful/total crosses (n)	Rho	0.405	0.405	0.115	-0.041	-0.207	-0.308
	p	0.217	0.217	0.736	0.904	0.542	0.357
Successful assists (n)	Rho	-0.358	-0.075	-0.133	-0.243	0.058	-0.168
	p	0.279	0.826	0.697	0.472	0.866	0.622
Total assists (n)	Rho	-0.182	-0.300	-0.364	-0.245	0.618*	-0.445
	p	0.593	0.370	0.272	0.467	**0.043**	0.170
Successful/total assists (n)	Rho	-0.358	-0.017	-0.006	-0.092	-0.012	-0.006
	p	0.279	0.960	0.987	0.787	0.973	0.987
Shots towards goal (n)	Rho	-0.573	-0.682*	-0.545	0.200	0.700*	-0.418
	p	0.066	**0.021**	0.083	0.555	**0.016**	0.201
Total shots (n)	Rho	-0.237	-0.346	-0.296	0.228	0.346	-0.424
	p	0.483	0.297	0.377	0.501	0.297	0.194
Shots towards goal/total shots (n)	Rho	0.155	0.136	0.000	-0.091	-0.027	0.218
	p	0.650	0.689	1.000	0.790	0.937	0.519
Ball possession (sec)	Rho	-0.300	-0.373	-0.573	-0.582	0.655*	-0.627*
	p	0.370	0.259	0.066	0.060	**0.029**	**0.039**

*($p \leq 0.05$),**($p \leq 0.01$) significant correlations.
