# Supplementary material for: Technical, Tactical, and Time–Motion Match Profiles of the Forwards, Midfielders, and Defenders of a Men’s Football Serie A Team
Source: Sports (Basel). 2025 Jan 21;13(2):28. doi: 10.3390/sports13020028 (PMC11860454; doi:10.3390/sports13020028)
Supplement: Supplementary file 1 [file sports-13-00028-s001.zip › Table S3.pdf]

Table S3. Spearman (Rho and significance values) correlations between TMA and technical and tactical indicators for defenders.

Indicators	Correlations	TD	Z2	Z3	Z4	MPErec	Burst
Played balls (n)	Rho	0.350	0.150	0.183	0.133	0.317	-0.733*
	p	0.356	0.700	0.637	0.732	0.406	**0.025**
Successful passes (n)	Rho	-0.150	-0.267	-0.367	-0.350	0.367	-0.817**
	p	0.700	0.488	0.332	0.356	0.332	**0.007**
Successful playing patterns (n)	Rho	0.517	0.267	0.233	0.233	0.150	-0.500
	p	0.154	0.488	0.546	0.546	0.700	0.170
Lost balls (n)	Rho	0.733*	0.500	0.600	0.600	0.033	-0.200
	p	**0.025**	0.170	0.088	0.088	0.932	0.606
Fouls committed (n)	Rho	-0.133	-0.183	-0.317	-0.100	0.350	-0.233
	p	0.732	0.637	0.406	0.798	0.356	0.546
Fouls received (n)	Rho	0.217	0.000	0.000	-0.017	-0.133	-0.233
	p	0.576	1.000	1.000	0.966	0.732	0.546
Successful dribbling (n)	Rho	0.831**	0.627	0.661	0.610	-0.034	-0.220
	p	**0.006**	0.071	0.053	0.081	0.931	0.569
Total dribbling (n)	Rho	0.862**	0.678*	0.686*	0.661	-0.033	-0.151
	p	**0.003**	**0.045**	**0.041**	0.053	0.932	0.699
Successful/total dribbling (n)	Rho	0.630	0.341	0.289	0.077	-0.060	-0.281
	p	0.069	0.370	0.450	0.845	0.879	0.464
Successful crosses (n)	Rho	0.836**	0.679*	0.714*	0.531	0.165	-0.435
	p	**0.005**	**0.044**	**0.031**	0.141	0.671	0.242
Total crosses (n)	Rho	0.485	0.527	0.343	0.159	0.033	-0.243
	p	0.185	0.145	0.366	0.683	0.932	0.529
Successful/total crosses (n)	Rho	0.621	0.456	0.511	0.201	-0.183	-0.128
	p	0.074	0.217	0.160	0.604	0.638	0.743
Successful assists (n)	Rho	0.548	0.456	0.548	0.342	-0.068	0.068
	p	0.127	0.217	0.127	0.367	0.861	0.861
Total assists (n)	Rho	0.683*	0.650	0.583	0.533	-0.183	-0.283
	p	**0.042**	0.058	0.099	0.139	0.637	0.460
Successful/total assists (n)	Rho	0.548	0.456	0.548	0.342	-0.068	0.068
	p	0.127	0.217	0.127	0.367	0.861	0.861
Shots towards goal (n)	Rho	0.837**	0.611	0.644	0.469	0.092	-0.293
	p	**0.005**	0.081	0.061	0.203	0.814	0.444
Total shots (n)	Rho	0.837**	0.611	0.678*	0.552	0.209	-0.360
	p	**0.005**	0.081	**0.045**	0.123	0.589	0.342
Shots towards goal/total shots (n)	Rho	0.378	0.042	0.101	-0.143	0.244	-0.395
	p	0.316	0.915	0.796	0.714	0.527	0.293
Ball possession (sec)	Rho	-0.300	-0.533	-0.617	-0.367	0.567	-0.650
	p	0.433	0.139	0.077	0.332	0.112	0.058

*($p \leq 0.05$),**($p \leq 0.01$) significant correlations.
